# Modulating Expression Levels of TCP Transcription Factors by *Mentha x piperita* Volatiles—An Allelopathic Tool to Influence Leaf Growth?

**DOI:** 10.3390/plants11223078

**Published:** 2022-11-14

**Authors:** Matthias Preusche, Marvin Vahl, Johanna Riediger, Andreas Ulbrich, Margot Schulz

**Affiliations:** 1 Department of Horticultural Production, University of Applied Science, 49090 Osnabrück, Germany; 2 Institute of Molecular Physiology and Biotechnology of Plants (IMBIO), University of Bonn, 53127 Bonn, Germany

**Keywords:** *Mentha x piperita* volatiles, *Brassica oleracea*, *Arabidopsis thaliana*, TCP transcription factors, leaf growth, fumigation

## Abstract

Peppermint (*Mentha x piperita*) is a species with inhibitory allelopathic properties due to its high amounts of terpenes. Recent studies have disclosed dosage dependent growth promotion or defense reactions in plants when facing appropriate amounts of *Mentha* bouquet terpenes. These positive effects could be of interest for agricultural applications. To obtain more insights into leaf growth modulations, the expression of *Arabidopsis and Brassica rapa* TCP transcription factors were studied after fumigation with *M. x piperita* bouquets (*Arabidopsis*), with *M. x piperita* essential oil or with limonene (*Arabidopsis* and Chinese cabbage). According to qPCR studies, expression of *TCP3*, *TCP24*, and *TCP20* were downregulated by all treatments in *Arabidopsis*, leading to altered leaf growth. Expressions of *B. rapa TCPs* after fumigation with the essential oil or limonene were less affected. Extensive greenhouse and polytunnel trials with white cabbage and *Mentha* plants showed that the developmental stage of the leaves, the dosage, and the fumigation time are of crucial importance for changed fresh and dry weights. Although further research is needed, the study may contribute to a more intensive utilization of ecologically friendly and species diversity conservation and positive allelopathic interactions in future agricultural systems.

## 1. Introduction

Terpenoids, one of the largest groups of plant and microbe secondary metabolites, can positively or negatively impact seed germination and plant growth. Depending on the dosage, many terpenoids have phytotoxic properties [1]. For instance, farnesene, a sesquiterpene, influences cell division, abolishes gravitropism in *Arabidopsis*, and downregulates auxin polar transport proteins [2]. Monoterpenes are described as interacting with transcriptional activities, cell division, and cytoskeletal elements in fumigated receiver plants, which results in growth inhibition or even plant death [3,4,5,6,7]. Volatile bouquets emitted from aromatic plants, such as *Mentha x piperita*, affect the transpiration of neighbored *Arabidopsis* plants by inducing stomatal opening [7]. Therefore, terpenes are considered as lead structures for the design of new herbicides. However, the effects of terpenoids are dosage dependent. Consistently, low dosages can have plant growth promoting properties. 

*Mentha x piperita* L. belongs to the Lamiaceae, a family with numerous species containing high amounts of essential oil with antifungal, bactericidal, insecticidal, and antifeedant properties. More than 50 constituents have been identified in the essential oil fraction [8], which can quantitatively and qualitatively vary from cultivar to cultivar and from leaf to leaf. The composition is determined by genetic factors, depending on culture methods, plant age and organ, microbial interactions, further interkingdom communication, and other biotic and abiotic parameters such as climate, geographical region, altitude, and irradiation. The most abundant compounds in the essential oil fraction and in the bouquets emitted by *Mentha x piperita* are limonene, linalool, menthone, menthol, and pulegone, while the concentration of single compounds in the emitted bouquets differ from the essential oil fraction of the leaves [9]. Limonene is a precursor of many other monoterpenoids, such as pulegone, menthol, and menthone, and its contents vary over the course of the season [10,11]. 

In a field study, Sukegawa et al. [9] reported the strong upregulation of *Glycine max* (soybean) defense genes in the leaves when plants are exposed to *Mentha x piperita* volatiles. They found a lower damage of *Brassica rapa* by herbivores when co-cultured with peppermint. We found in a previous study that the volatile bouquet of *Mentha x piperita* (English Mint cv. Mitcham) positively influenced leaf development in young white cabbage (*Brassica oleracea* convar. *capitata* var. *alba*) plants during a 27-day-fumigation, while the influence on leaf growth seemed to depend on responsiveness during early stages of leaf development. A changed leaf shape but no enhanced leaf growth or dry weight was seen when young plants were exposed to menthol or to a combination of menthol and menthone [12]. The observation that the youngest leaves were sensitive to the treatment led to the assumption that fumigation with the *Mentha* volatile bouquet entails modulations of fundamental processes in early leaf development, which results in an enhanced growth of the young leaf. Thus, the responsiveness of transcription factors, in particular of TCPs, to *Mentha x piperita* monoterpenes could be causative for the modulation of leaf growth and development.

The differentiation process in the developing leaf is regulated by a network of hormones and transcription factors, while TCPs (TEOSINTEBRANCHED1/CYCLOIDEA/PROLIFERATING CELL FACTOR) are of particular importance. The roles and interactions of TCPs have been studied intensively during the last few decades [13,14], but many aspects of the complicated, hierarchically organized network with counteracting and redundant functions in controlling plant developmental processes are still elusive [15,16]. 

The TCP family is composed of two different classes, class I and class II. In *Arabidopsis thaliana*, the class II subfamily is further divided into the subclasses CYC/TB1 and CIN. While being under the control of miRNA 319 (miR319), TCP2, TCP3, TCP4, TCP5, TCP10, TCP13, TCP17, and TCP24 regulate leaf development. The miR319 interaction site is located near the 3′ part of the coding region and not within the *TCP* domain, a characteristic region of the *TCP*s [17]. This TCP domain is necessary for protein–protein interactions [18]. The miR319 interaction site is of importance for class II TCP mRNA abundance, as downregulation of class II TCPs seems to be regulated by miRNA-mediated degradation of their mRNAs [19]. Thus, high levels of miR319 downregulate these TCPs with effects on leaf morphogenesis in *Arabidopsis* [20].

Another group of TCPs have genes lacking the miRNA319 target sequence. The non-miR319-regulated class I TCPs, for instance TCP20 and TCP9, inhibit lipoxygenase 2 gene (*LOX2*) expression, which is crucial for jasmonate biosynthesis. *LOX2* expression is thought to be regulated by the ratios of class I and class II TCP (TCP4) levels [21]. While members of class II TCPs inhibit growth cell division and promote aging, members of class I TCPs have the opposite functions by controlling the expression of specific target genes [22,23]. Non-miR319-regulated TCP20 and TCP9 are also reported to control senescence. TCPs of the same class form homo- or hetero-dimers, which increases the flexibility in interacting with other proteins [19]. The TCP/miRNA interactions, the complex, not yet completely understood regulation processes by TCPs, their known target genes, as well as interaction with plant hormones and jasmonate synthesis, influences of light, temperature, and further factors on TCPs, have recently been reviewed [13]. 

In this study, the influence of *Mentha* volatiles on the gene expressions of CIN branch TCPs (TCP2, TCP3, TCP4, TCP10, and TCP24), the non-miR319-regulated class 1 TCP 20 and TCP9, and *LOX2* were investigated using the model plant *Arabidopsis thaliana*. These studies were performed to obtain clues concerning the essential interferences of the volatiles with fundamental processes in leaf development after 1, 3, and 7 days of fumigation. The expression of well-known TCP genes of *Brassica rapa* ssp. *Pekinensis*, [24,25] was studied after 7 days of fumigation. The major part of our study deals with the impacts of *Mentha piperita* bouquet volatiles on white cabbage plants in greenhouse and polytunnel trials to reveal conditions that may allow an ecologically acceptable utilization of a positive allelopathic interaction of *Mentha* bouquet terpenoids for the modulation of leaf development in horticulture.

## 2. Results

### 2.1. Effects of Fumigations on the Arabidopsis Phenotype

**Figure 1:**
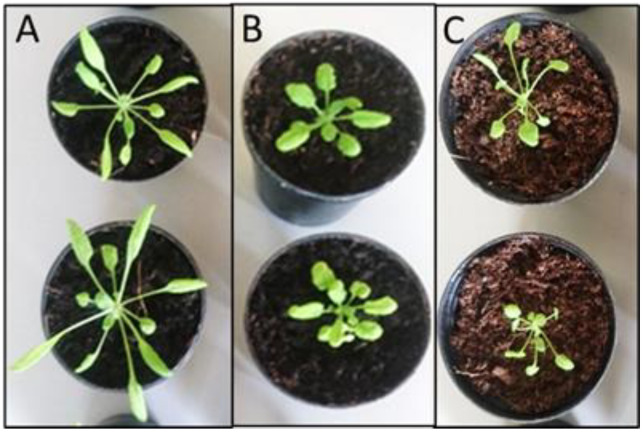
Effects of volatile bouquets emitted from different sized *Mentha* plants on the phenotype of *Arabidopsis thaliana* Col-0. (**A**): *Mentha* plant of 10 shoots and 3 nodes; (**B**): control; (**C**): *Mentha* plants of 10 shoots and 5 nodes. Fumigation time was one week.

Fumigation with one *Mentha* plant of 10 shoots and 3 nodes or with larger plants possessing 5 nodes in the growth chamber severely altered the phenotype of *Arabidopsis* plants in comparison to the control plants. In the presence of the smaller *Mentha* plants, *Arabidopsis* developed larger rosettes with longer narrow leaves and longer petioles. A similar result was obtained with *Mentha* essential oil. Limonene led to longer petioles, whereas the leaf shape was not changed. With larger *Mentha* plants, the growth of *Arabidopsis* was heavily reduced (Figure 1), indicating a strong, negative influence of higher volatile concentrations. The negative growth effects are in agreement with the findings that many terpenes have phytotoxic properties when applied in high concentrations [1]. 

### 2.2. Determinations of White and Chinese Cabbage Fresh and Dry Weights after Fumigation with Limonene and Mentha Essential Oil

**Figure 2:**
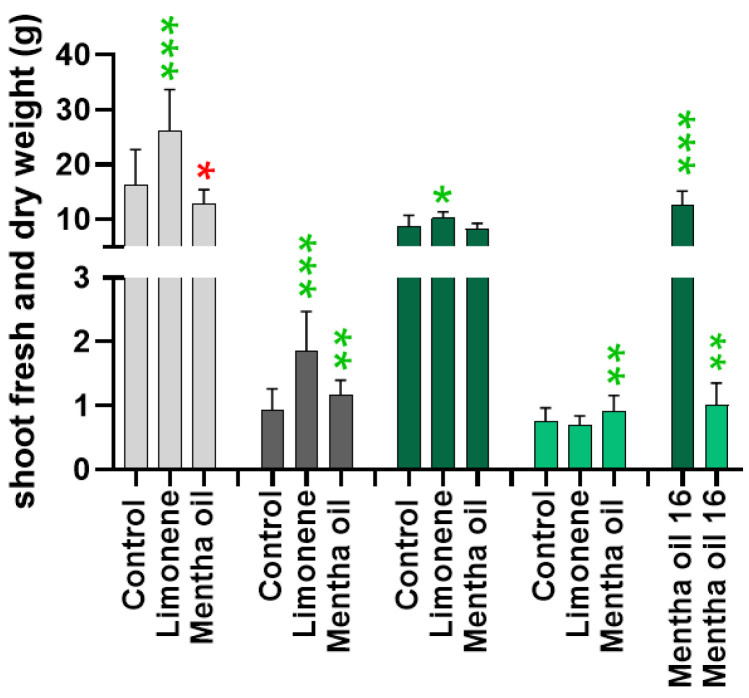
Fresh (FW) and dry weight (DW) of above-ground plant material after 1-week-fumigation in the greenhouse with high concentrations of limonene (8 mL/day) and *Mentha* essential oil (14 or 16 mL/day) and controls. White cabbage: light grey (FW), dark grey (DW); Chinese cabbage: dark green (FW), green (DW). *Mentha* oil 16 indicates 16 mL/day, for all other treatments 14 mL/day were used. Significance: * *p* < 0.05; ** *p* < 0.005; *** *p* < 0.0005), green asterisks: increase, red asterisk: reduction.

Fresh and dry weights of the above-ground parts of white and Chinese cabbage plants were determined after fumigation with limonene or *Mentha* essential oil for one week to estimate threshold ranges of terpenoid concentrations causing inhibitory effects on cabbage growth. In contrast to *Arabidopsis*, no striking alterations of the phenotypes were observed. Nevertheless, white cabbage developed a significantly higher fresh and dry weight with limonene and a higher dry weight with *Mentha* essential oil, but a slightly reduced fresh weight (Figure 2). Regarding the fresh weight, Chinese cabbage was less responsive than white cabbage to limonene, although dry weight was also significantly enhanced. Here, *Mentha* essential oil increased fresh weight with high significance only with the 16 mL dosage. 

### 2.3. TCP Transcription Factors—Targets of Mentha x piperita Emitted Volatiles, Essential Oil Fraction, and Limonene

**Figure 3:**
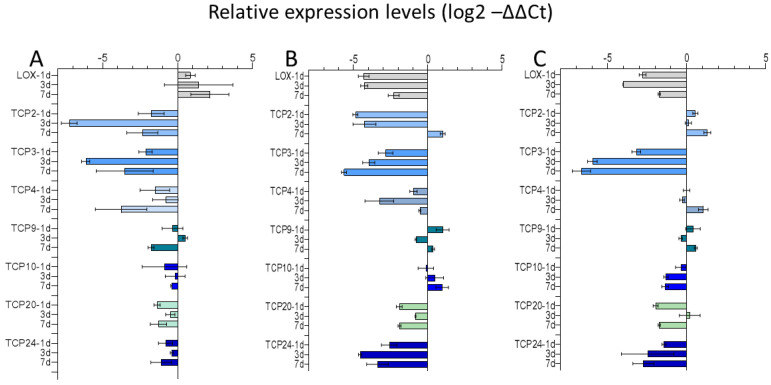
TCP gene expression in *Arabidopsis* is modulated by *Mentha* volatiles. Expression of class II *TCP2*, *TCP3*, *TCP4*, *TCP5*, *TCP10*, *TCP13*, *TCP17*, *TCP24*, and class I *TCP20* and *TCP9* genes, *LOX2* (LOX) after the different types of fumigation. **A**: *Mentha* plant; **B**: *Mentha* essential oil; **C**: limonene. The figure shows mean values and standard error of means (n = 2 samples, each sample prepared from 43–51 plants per time point).

Because it was intended to address the growth promotion and phenotype modeling of *Arabidopsis* by *Mentha* volatile bouquets, TCP gene expressions were investigated with plants fumigated with the smaller *Mentha* plants. qPCR analysis with cDNA from growth-promoted *Arabidopsis* plants revealed a strong downregulation of *TCP2* and *TCP3* transcripts, most exhibited after 3 days of fumigation, and a downregulation of *TCP4*, strongest at day 7, while *TCP 9*, *TCP10*, *TCP 20*, and *TCP24* transcript levels showed a much weaker response and were only slightly, if at all, downregulated (Figure 3). The *LOX2* gene expression was ambivalent but tended towards an upregulation at day 7 without significance. Fumigation with the essential oil fraction led to similar results, i.e., a downregulation of *TCP2* transcripts after 1 and 3 day(s) and a slight upregulation at day 7. *TCP3* mRNA was downregulated at all time points. A weaker response of *TCP4* transcripts was stated, with the highest downregulation at day 3. Again, *TCP 9* and *TCP10* transcripts were only slightly affected, with a low upregulation of *TCP9* at day 1 and of *TCP10* at day 7, while *TCP20* and in particular *TCP24* were more strongly downregulated than was found after *Mentha* bouquet fumigation. The *LOX2* transcripts were downregulated. Using limonene for fumigation resulted in similar transcript profiles for *TCP3, TCP9*, and *TCP20*; *TCP10* was downregulated, but *TCP2* and *TCP4* were almost not affected. The downregulation of *TCP24* was between the one found for the bouquet and essential oil fumigation. Thus, fumigation with limonene had almost no effect on transcripts of *TCP2* and *TCP4*, which were responsive to the complex mixture of the volatiles present in the bouquet and the essential oil. The *LOX2* transcript level was similarly affected, as found after the essential oil fumigation. Most of the expression profiles showed a dependence on the fumigation time, in particular *TCP2*, *TCP3*, *TCP4*, and *LOX2* transcript abundances.

#### 2.3.1. Expression of TCP Transcription Factors in Chinese Cabbage after Exposure to Mentha Essential Oil Fraction and Limonene

**Figure 4:**
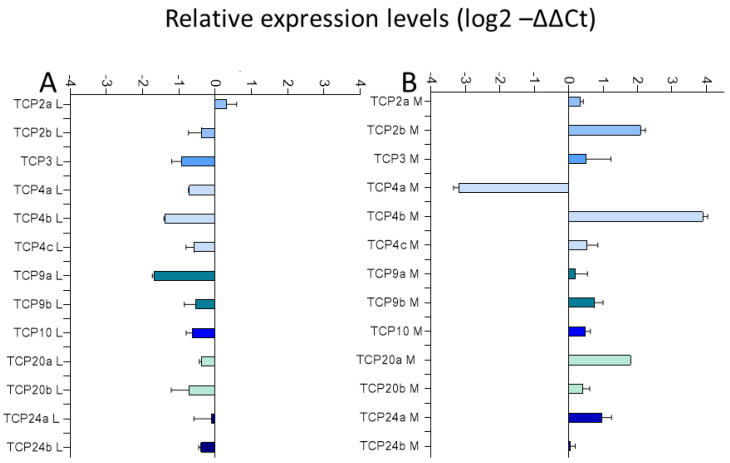
Expression of *Brassica rapa* ssp. *Pekinensis* TCP transcription factors in response to *Mentha x piperita* essential oil and limonene. **A**: limonene; **B**: *Mentha* essential oil. The figure shows mean values and standard error of means (n = 2 composed samples).

In contrast to *Arabidopsis thaliana*, Chinese cabbage has two *BraTCP2*, one *BraTCP3*, three *BraTCP4*, one *BraTCP10*, and two *BraTCP24* genes [24,25]. When fumigated with the *Mentha* essential oil fraction or limonene, expressions of the tested *Brassica rapa* ssp. *Pekinensis* TCP transcription factors were considerably less affected than the *TCPs* of *Arabidopsis*. With limonene, all *TCP*s were downregulated in the rage of −0.5 to −2fold, except for *TCP2a*, which was slightly upregulated, and *TCP24a*, which was not responsive. With the essential oil fraction, the only downregulated TCP was *TCP4a* (−3 fold), while *TCP4b* was 4-fold, but *TCP4c* was only 0.5-fold upregulated (Figure 4). The latter range of upregulation was also found for *TCP2a*, *TCP3*, *TCP9a*, *TCP10*, and *TCP20b*. *TCP24b* was not responsive, and *TCP2b* showed a 2-fold, *TCP9b* and *TCP24a* each showed a 1-fold, and *TCP20a* showed an approximate 2-fold upregulation.

#### 2.3.2. Choice of White Cabbage for Greenhouse and Polytunnel Trials

In turnips (*Brassica rapa* ssp. *rapa*), *Brr/BraTCP2* is involved in leaf development by regulating cell proliferation [25]. According to Efroni et al. [26], CIN-class TCPs have crucial functions, such as being negative regulators of cell proliferation, therefore causing leaf differentiation [24]. Figure 2 and Figure 4 illustrate that the fumigation with limonene and *Mentha x piperita* essential oil have different impacts on (1) leaf fresh and dry weight and (2) TCP gene expressions. With Chinese cabbage, the study with *Mentha* essential oil disclosed that head shape modulating TCP4a was the only downregulated TCP gene; all others were upregulated or not responsive. Because downregulation of the CIN-class TCP abundance is a prerequisite for cell proliferation, enhanced leaf growth of Chinese cabbage may not occur when the plants were fumigated with *Mentha* plants as the fresh and dry weight determinations after limonene and *Mentha* essential oil fumigation indicated that Chinese cabbage needs higher volatile concentrations than white cabbage to achieve growth responses. Thus, the concentrations of *Mentha* bouquet compounds may not be high enough for modulating Chinese cabbage leaf growth, but could be high enough for white cabbage. Therefore, and by considering the results of the former pilot study [12], fumigation with *Mentha* plants in greenhouse and polytunnel experiments were performed with white cabbage and not with Chinese cabbage. The responsiveness of TCP transcription factors towards *Mentha* volatiles found for *Arabidopsis* was assumed to be similar in white cabbage.

### 2.4. Greenhouse and Polytunnel Trials with Brassica oleracea convar. capitata var. alba and Mentha plants

#### Greenhouse Trial—Leaf Length and Width, Growth Increments

Leaf length and width were measured after 1 (T1), 4 (T2), 8 (T3), 11 (T4), and 15 days (T5) after exposure to *Mentha* volatiles emitted by one or four plants (Figure S1). At T1 and T2, where only the first and the emerging (T1) or the further developed (T2) second leaves were present, growth showed no significant differences to the controls. At T3 (8 days), the difference in leaf length of control plants and those exposed to four *Mentha* plants (4M) was significant larger for leaf 2 when the distance to the *Mentha* plants was more than 35 cm. Leaf 1 was not responsive and leaf 3 tended towards an increased length growth but without significance. Leaf width was not affected at T3. The largest differences were seen with leaf 2 and leaf 4, but particularly with leaf 3 after 15 days (T5) when plants were exposed to four *Mentha* plants when the distance to the *Mentha* plants was more than 20 cm. Thus, the number of *Mentha* plants, dependence on the distance, and the dosage are crucial for leaf parameter modulations. The results indicate again that defined developmental stages of a leaf in combination with the dosage of the volatiles are important for the response to the treatments. To summarize, only the second and third leaves at T3, T4, and T5, and also the fourth leaf, clearly responded to the treatment with bouquet volatiles emitted from four *Mentha* plants. The highest increases in leaf length and width were obtained at distances exceeding 35 cm. 

**Figure 5:**
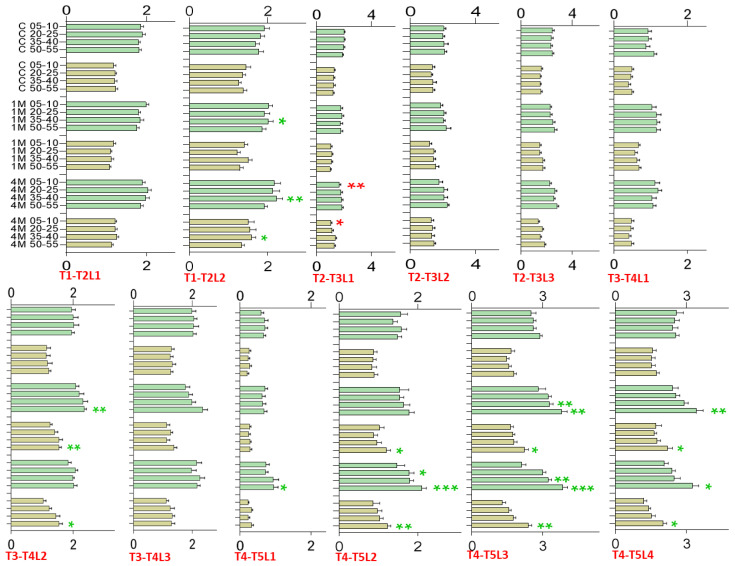
Greenhouse Trial: Increments (cm y-axis, consider graph right 90° rotation) in length (green) and width (olive) of the first two to four leaves (T1–T2, T2–T3, T3–T4, and T4–T5) at four difference distances (0.5–10, 20–25, 35–40 and 50–55 cm, x-axis, consider graph right 90° rotation) in control plants (no *Mentha*), plants exposed to one *Mentha* (1 M), and plants exposed to four *Mentha* (4 M) plants. Green asterisks indicate significantly increased growth, red asterisk significant lower growth compared to the controls, bars without asterisk: no significance. Enhanced increments occurred predominantly during the late period of fumigation with leaf 2 (T3–T4) and with leaves 2, 3, and 4 at T4–T5. The figure shows mean values and standard error of means (n = 16). Significance with reference to the appropriate cm control (no fumigation), (*t*-test): * *p* < 0.05; ** *p* < 0.005; *** *p* < 0.0005). Red labels give the increment (T1–T2, T2–T3, T3–T4, and T4–T5) and the leaf number (L1, L2, L3, L4).

Measurements of the leaf increments support the observations (Figure 5). Compared to the controls, leaf growth increments show that the responses of leaves 2, 3, and 4 to the *Mentha* volatiles are significantly higher between 11 and 15 days (T4–T5) of fumigation when the distance to four *Mentha* plants was more than 25 cm. Increments of fumigated leaves at T1–T2, except for leaf 2, and T2–T3 were similar to the control values. The increments of leaf 2 were continuously larger after 8 days of fumigation but not during the early phase of the treatment.

### 2.5. Polytunnel Trial—Leaf Length and Width, Growth Increments

Leaf length and width were measured after 1 (T1), 4 (T2), 8 (T3), 12 (T4), and 15 days (T5) after exposure of the plants to volatiles emitted by four or nine *Mentha* plants. At all time-points, leaf 1 was comparable to the control, thus without response to the treatment. At T2, T3, and T4, the length growth of leaf 2 and, to a lesser degree, leaf 3 were enhanced by the bouquets of four and nine mint plants at both distances. At T5, the growth promoting effect of the volatiles was almost halted in leaf 2 and leaf 3. Leaf width was enhanced only at T3 with high significance and again only in leaf 2 and leaf 3. Thus, for leaf 2 and leaf 3, length growth was more affected than width growth and overall responsiveness, mainly restricted to leaf 2 and leaf 3, occurred between day 4 and day 12 of the treatment (Figure S2).

**Figure 6:**
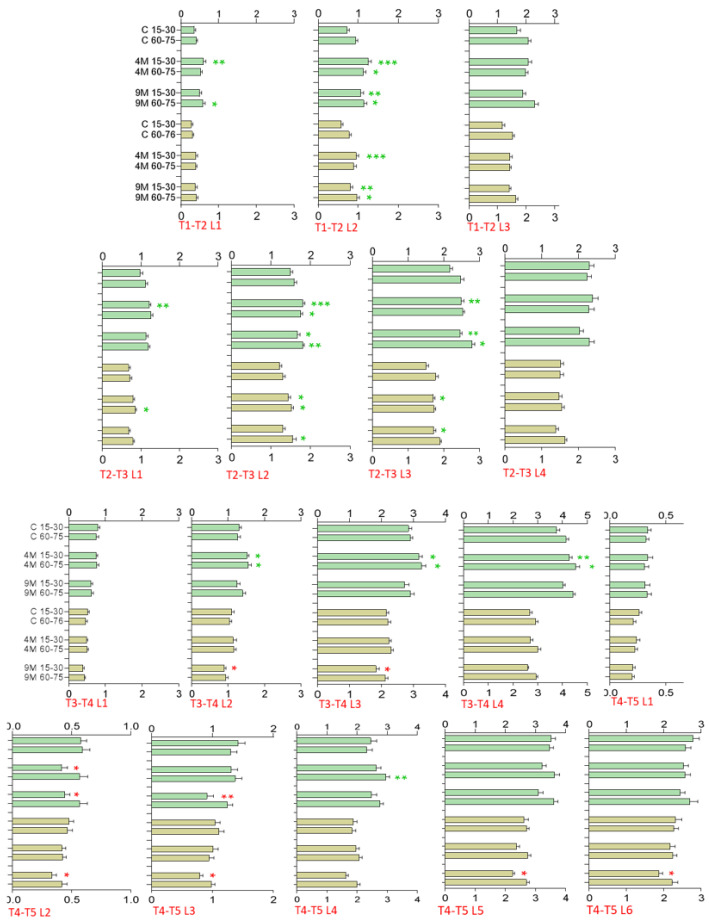
Polytunnel Trial: Consider graph right 90^o^ rotation. Increments in length (green) and width (olive) of the first three to four leaves (T1–T2, T2–T3, T3–T4, T4–T5) at two difference distances (15–30 cm and 60–75 cm) in control plants (no *Mentha*), plants exposed to four *Mentha* (4 M), and plants exposed to nine *Mentha* (9 M) plants. Green asterisks indicate significantly increased growth, red asterisks indicates significantly lower growth compared to the controls, bars without asterisk: no significance; n= 24. Enhanced growth is primarily found during the early period of fumigation with leaves 2 and 3, while during the late period, a significantly inhibited or at least a reduced growth of these leaves was predominately seen. Significance with reference to the appropriate cm control (no fumigation), (*t*-test): * *p* < 0.05; ** *p* < 0.005; *** *p* < 0.0005). Red labels give the increment (T1–T2, T2–T3, T3–T4, and T4–T5) and the leaf number (L1, L2, L3, L4, L5, L6).

The increments of leaf growth, depictured in Figure 6, illustrate the dynamics of leaf growth more distinctly. Increments of leaves 2 and 3 occurred mainly between T1–T2 (leaf 2) and T2–T3 (both leaves), while at T3–T4, only four mints had a positive effect on leaf length at both distances. Leaf width did not increase and was even reduced at the short distance with nine *Mentha* plants. The reduction in increments, compared to control values or even lower values, continued at T4–T5 for all leaves (except for leaf 4) with four mint plants at the larger distance (60–75 cm). The increments demonstrate that the growth promoting effect of *Mentha* bouquet affected leaves in defined developmental stages only temporarily. No growth promotion was found with leaves that were developed during the late period of the treatment (T5, leaves 4, 5, and 6). 

### 2.6. Greenhous and Polytunnel Trials—Fresh and Dry Weight

**Figure 7:**
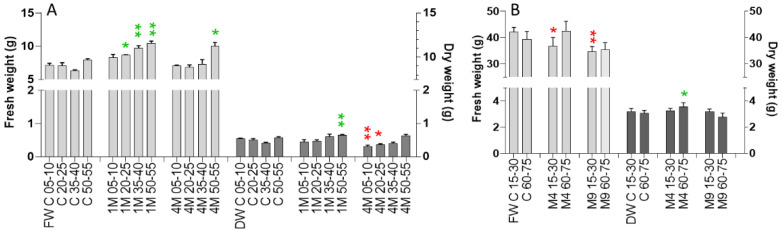
(**A**): Greenhouse trial: Fresh and dry weights after termination of the treatment (means + standard error of means). (**B**): Polytunnel Trial: Fresh and dry weights after termination of treatment (means + standard error of means). Light grey bars: fresh weight; dark grey bark dry weight. Green asterisks indicate increase, red asterisk inhibition, significance levels: (*t*-test): * *p* < 0.05; ** *p* < 0.005); bars without asterisk: no significance. Notice that the number of plants used for fresh and dry weight determinations was different compared to the greenhouse trial, due to the different arrangements.

The fresh and dry weight of the above-ground plant material was determined after termination of the fumigation at day 21. Fresh and dry weights of the cabbage plants cultured in the greenhouse were enhanced when the distance to one mint was more than 35 cm; with four mint plants, more than 50 cm were necessary to achieve a similar increase (Figure 7). Four *Mentha* plants led to lower dry masses at the smaller distances. Fresh and dry weights of the entire above-ground plant material mirror the overall growth, while increases must be attributed mainly to the second and third leaves. Nevertheless, the total increase of above-ground biomass fresh weight was in the range of 20% (calculated from mean values) of the above controls for plants growing with a 35–55 cm distance to one *Mentha* plant. The total dry weight was lower than control values, with the exception of the plants growing with a distance of 55 cm to one *Mentha* plant. Here, the increase in dry weight was approximately 10%.

As a consequence of the more or less stagnating leaf growth during the late period of the treatment (T5, 15 days) in the polytunnel trial, no enhanced fresh and dry weights of the above-ground plant material was found after treatment termination (21 days) (Figure 7). The cabbage plants cultured in the polytunnel developed more leaves compared to the plants in the greenhouse, due to the different culture conditions (pre-culture in the greenhouse in pots prior the planting in the polytunnel soil). Leaves 7 and 8 were very small and did not considerably contribute to fresh and dry weights. Because of their small size, these leaves were not evaluated further. Taken together, a higher biomass due to the volatile promoted growth of leaves 2 and 3 at T2, T3, and T4 was not a decisive factor when the fresh and dry weights of the above-ground biomass (Figure 7) was determined after 21 days. In comparison to the greenhouse trial with the strongest effects between 11 and 15 days (T4–T5), growth promoting effects occurred much earlier, namely between 4 and 12 days, when the plants were cultured in the polytunnel with 4 and 9 *Mentha* plants and was characterized by a subsequent decline (Figure 5 and Figure 6).

## 3. Discussion

Presently, approaches to integrate positive allelopathic interactions in agricultural practices are rarely addressed, although the positive effects of many allelochemicals when applied in low concentrations are well known. This study disclosed some of the difficulties associated with the suitable application and maintenance of plant growth promoting concentrations of *Mentha x piperita* emitted volatiles and, on the other side, the dynamics in responses of the receiver plants. 

The involvement of TCPs indicates that dosage dependent responsiveness to *Mentha* volatiles starts on superordinated levels that regulate the leaf growth process and is embedded in the entire life cycle of the plant. Causative molecular interactions that lead to modulations of transcriptional activities, resulting perhaps in shifts of the ratios of class I and class II TCP levels, could not be elucidated in this study, nor could the responsiveness of TCP target genes and hormone interactions or levels of miR319. Nevertheless, for a possible interpretation of the results, we would like to address some of the far-reaching downstream events of gene activity cascades that are presumably linked to *Mentha* volatile induced modulations of TCP transcript abundances.

Treatments with limonene, *Mentha* essential oil, and *Mentha* bouquet all downregulated *TCP3*, *TCP24* (both class II CIN branch TCPs), and *TCP20*, a class I non-miR319-regulated TCP in *Arabidopsis*. TCP3 negatively affects auxin responses [27]. Auxin is known to be crucial in leaf development for its functions in leaf initiation and influences on leaf size, shape, and form [28,29]. The effects of TCP3 on the auxin response and on flavonoid biosynthesis have been investigated by Li and Zachgo [30]. Thus, downregulation of TCP3 expression might have reverse effects on auxin triggered processes that are under the control of TCP3. Downregulation of TCP3 by *Mentha* volatiles should therefore influence effects linked with auxin responses. *TCP20* is another partner in the auxin network as it regulates the expression of *GH3.3*. GH3s are known to control the formation of IAA-amino acid conjugates, which are important in maintaining auxin homeostasis [31]. In our study, downregulation of TCP20 gene expression was, however, found to be less severe than that of *TCP3*. *TCP24* suppresses secondary wall thickening [32]; thus, a downregulation of *TCP24* transcription can lead to enhanced secondary cell wall formation, which would be in line with the increased dry weights of white cabbage after fumigation with high amounts of *Mentha* volatiles. Another group of transcription factors (growth-regulating factors (GRFs), which is under the control of miR396 [33], might also be affected, but the study of their expression profiles after *Mentha* fumigation remains to be carried out.

The results indicate that the positive effects of *Mentha* volatiles on leaf development probably proceed first from the interactions of volatile components with TCPs. Growth stimulation may later be due to interactions with the auxin network. Other downstream interactions with hormones and proteins are presumable, but further investigations must prove the assumption. The many antagonistic and synergistic interactions and functional redundancies raise difficulties in unambiguous assignments of the observed effects to defined developmental pathways. In addition, alterations in downstream events, performed as counteracting steps by the plant or its associated microorganisms, for instance monoterpene detoxification, may intercept responses to the volatiles.

Down regulation of the gene expression of other transcription factors, such as MYC-2, ANAC, and SCR-SHR, has been found after the application of the monoterpene citral. A binding of citral to these and further transcription factors (SSBPs WHY1, WHY2, and WHY3) was concluded from in silico molecular docking analyses [3]. Godard et al. [34] reported myrcene or ocimene induced expression of transcription factors, stress, and defense genes in *Arabidopsis*. The gene expression, at least of several plant transcription factors but also of some genes involved in defense, is apparently sensitive to terpenoid volatiles. Thus, it is possible that interactions of terpenoids with transcription factors and perhaps with many other proteins are common features. With MYC-2, ANAC, SCR-SHR, SSBPs WHY1, WHY2, and WHY3, the molecular backgrounds of the modulation are also not yet understood. Sukegawa et al. [11] concluded that there was a fumigation-time dependent epigenetic regulation of the JA- and SA-signaling-dependent defense genes TI and PR1 by *Mentha x piperita* volatiles in soybean. Here, the transient chromatin acetylation levels of the regulatory promoter region of these genes were immediately changed after starting the fumigation [11]. It cannot be discounted that epigenetic regulation of volatile binding is important at certain stages of the plant’s life cycle, allowing defined gene responses or hindering them, leading to different gene expression profiles and finally to other protein interactions [35]. Some TCPs also seem to be under circadian clock control [36]; thus, transcriptional responses upon fumigation may succumb to diurnal variations. Transient changes in the gene expressions of the TCPs, in combination with the leaf life cycle and the abundance of miR319, and perhaps also epigenetic regulations, could be one reason for the decrease of the growth promoting effect of *Mentha* volatiles found with the plants grown in the polytunnel during the late phase of fumigation and may explain the failed growth stimulation in oldest leaves. 

Further explanations are given by the dosage effects. The greenhouse and the polytunnel trials indicate the possibility of transient TCP-involved interactions. Concentrations of volatiles able to elicit growth effects were reached earlier with a higher number of *Mentha* plants, and thus the threshold concentration necessary for leaf growth induction in the greenhouse is later in place. Leaf surface can be influential, particularly wax layers. Monoterpenoids have been found to precipitate on wax layers and permeate them subsequently [7]; thus, local concentrations on the leaf may be spotted and increase during the course of fumigation. Because wax deposition on cabbage leaves was found to increase with aging [37], inhibitory amounts of terpenoids can finally accumulate in the wax layer, serving as a local source for the release of the molecules that infiltrate leaf cells underneath. We assume that plants with a thinner wax layer than cabbage, such as *Arabidopsis*, are more quickly affected by low monoterpene concentrations, which would explain the rapidly altered expression profiles of sensitive TCPs. More for cabbage than for *Arabidopsis*, the shape of the shoot and the positioning of the leaves are properties that influence the receiving of volatiles by leaves. Nevertheless, with the exception of *TCP24*, the expression profiles of affected TCPs were surprisingly similar in *Arabidopsis* when fumigated with small *Mentha* plants or with *Mentha* essential oil. Are some of the volatiles better trapped in the wax layers than others? In this case, subsequently different TCP gene expression profiles in cabbage would occur, if their expression is compound specific. For volatile trapping, the structure of the wax crystals, the wax composition, and immersed secondary metabolites, for instance flavonoids, may be of importance for species-dependent differences in the responsiveness to *Mentha* volatiles. In addition, many plant species have low quantities of di- and triterpenes, sometimes rare ones, deposited in their wax layers, including *Arabidopsis* and *Brassica oleracea* [38,39,40], indicating that wax layers are suitable sites for terpenoid deposition. At present, it is speculative to assume an involvement of TCPs, perhaps TCP4, in wax layer modulations and functions.

On the other hand, the amounts of volatiles emitted by the *Mentha* plants can vary as the terpenoid biosynthesis is modulated by many factors such as season, temperature, wind, leaf and whole plant age, growth of the plant, and, as important modulators, by soil and plant microbiomes. The temperature dependency of *Mentha x piperita* essential oil composition is known [41]. Menthol and menthone decrease at high temperature, menthylacetate and pulegone increase, and others, such as limonene, are not affected. Consistently, the maintenance of identical volatile compositions over time is unlikely. Altered volatile compositions can be an additional, important reason for shaping the leaf responsiveness. Alterations should be more presumable in the polytunnel trial, with consequences for the leaf growth, because, in this trial, temperature was not controlled.

In summary, the study disclosed the leaf growth promoting and dosage dependent effect of *Mentha* volatiles in cabbage and *Arabidopsis* at defined developmental stages. Greenhouse cultures may be more suitable for utilizing the positive effects of *Mentha* volatiles on the leaf growth of young cabbage plants, and also on other important vegetables. The maintenance of optimal volatile concentrations over time is, however, an essential problem as the concentration depends on air circulation and temperature as well as alterations in the bouquet composition, and is also due to the different chemical properties, such as the volatility, of the constituents.

Although optimization of culture and fumigation conditions are expected to be species dependent and need further extensive research, the ecologically and environmentally friendly utilization of allelopathic volatiles in suitable concentrations for leaf growth promotion seems to be feasible, when harvest times are adapted. The results presented here are in line with our former pilot study [12]. Moreover, the growth promoting effects of *Mentha* species, when applied in low amounts, have been described by Islam and Kato-Noguchi [42]. Finally, the use of plant volatile bouquets for plant growth promoting effects does not have the potential risk to animal health and the environment as may occur with high amounts of terpenoids [43]. 

## 4. Material and methods 

### 4.1. Plant Material

#### 4.1.1. *Mentha x piperita* Variety English Mint cv. “Mitcham”

For the fumigation of receiver plants, *Mentha x piperita* L. variety English Mint cv. “Mitcham” was used. The young plants were purchased from a local gardener and grown in a greenhouse (20/16 °C day/night) for approximately one and a half month until the start of the experiments. Two weeks after delivery, *Mentha* plants were propagated form cuttings. The uniformly-sized *Mentha* plants were grown for one month under the same conditions as described. The *Mentha* plants were cut again one week before being grown (4 main shoots with 5 nodes each, if not otherwise noted). 

#### 4.1.2. *Arabidopsis thaliana* Col-0

Seeds of *A. thaliana* Col-0 were surface sterilized and germinated in petri dishes with standard MS media and 1% sucrose (Murashige & Skoog, without any additives) under sterile conditions. After two days of stratification at 4 °C, the plates were transferred into a growth chamber for germination and growth at 23 °C, 16/8 h day/night. After 10 days, the young seedlings were transferred into small pots (Ø 5 cm) filled with water-saturated soil (“Seedlingsubstrat” from Klaasmann-Deilmann, Germany). Treatments were started one day after pricking. 

#### 4.1.3. *Brassica oleracea* cv. *capitata* var. *alba* convar. LENNOX F1 (white cabbage) and *Brassica rapa* (Chinese cabbage)

White cabbages (*Brassica oleracea* cv. *capitata* var. *alba* convar. LENNOX F1, Bejo Germany) were grown as described in [12]. In short, cabbages were sown in pressed peat pots (4.5 × 4 × 5 cm, cf. Figure 1) and germinated under 16/10 °C day/night conditions in a greenhouse and grown for 14–21 days until the second leaf was approximately 1.5–2 cm long. During this period, the plants were watered once a day. The substrate used for germination and growth was “Potground P” (Klaasmann-Deilmann, Germany). Chinese cabbages (*Brassica rapa* ssp. *Pekinensis*, Bilko F1, Bejo, Germany) were similarly cultured. 

### 4.2. Fumigation Experiments

#### 4.2.1. Fumigation of *Arabidopsis thaliana* with *Mentha x piperita* Mitcham

For determination of *TCP* gene expression profiles, standardized *Mentha* plants were used. *A. thaliana* seedlings were co-cultured with one *Mentha* plant of 10 shoots and 3 nodes or with larger plants possessing 5 nodes. *A. thaliana* control groups were cultured without *Mentha* plants. These pre-experiments revealed the growth promoting and phenotype modeling properties of smaller *Mentha* plants, while the larger plants led to growth inhibition of the co-cultured *Arabidopsis* plants (Figure 1). The following experiments were therefore performed with the smaller *Mentha* plants. Co-culturing with *A. thaliana* after one day of acclimation was performed for 1, 3, and 7 days in the growth chamber.

#### 4.2.2. Fumigation of *A. thaliana* and *Brassica* plants with Mentha essential oil and limonene for Gene Expression Experiments

**Figure 8:**
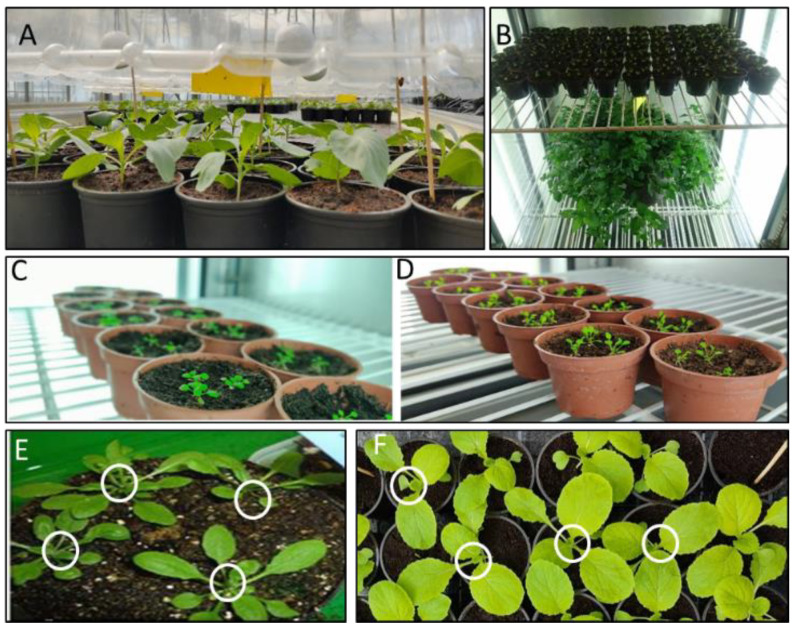
One-week-fumigation of cabbage and *Arabidopsis* for gene expression experiments. (**A**): Cabbage plants were fumigated with volatile-loaded cotton balls. Controls were with volatile-free cotton balls. (**B**): Fumigation arrangement of *Arabidopsis* with one *Mentha* plant in the growth chamber. (**C**): Control, (**D**): Limonene-fumigated *Arabidopsis* after 7 days. (**E**,**F**): Harvested shoot material for gene expression studies. The innermost parts of the *Arabidopsis* rosettes and shoot apex containing material of cabbage plants (white rings) were harvested and used for RNA extraction. Pictures (**E**) and (**F**) show control plants. (**E**): *Arabidopsis*; (**F**): Chinese cabbage.

**Figure 9:**
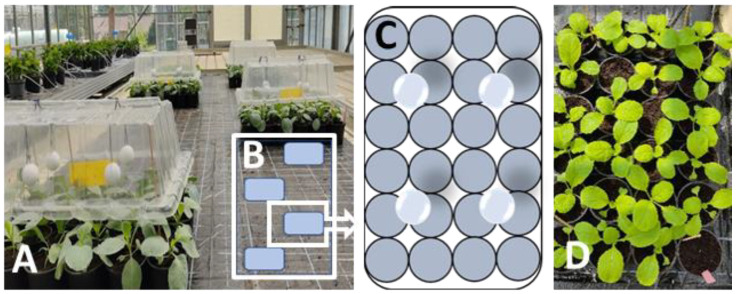
Experimental setup of cabbage plants in 4 culture boxes for limonene and *Mentha* essential oil fumigations in the greenhouse. Distances between culture boxes: 60 cm. (**A**,**C**): plants and cotton balls in one box ((**B**): inserted scheme in the picture of culture box arrangements in the greenhouse). (**D**): cabbage plants used for fresh and dry weight determination.

*A. thaliana* plants were treated with steam distillates of English mint essential oil, free of organic solvents (Norfolk Essential Oils, Wisbech, UK) or limonene (Merck Chemicals GmbH, Darmstadt, Germany; companies’ information regarding the products are given in the Supplementary File). Limonene is one of the known components of the *Mentha* essential oil fractions (in the used essential oil charge: 2.4%) and also of the plant-emitted bouquet. Limonene was also separately applied, because it is the precursor of further monoterpenes. The treatment was performed for one week by injecting the essential oil fraction or limonene into cotton balls swinging approximately 5 cm above the plants (Figure 8 and Figure 9).

These arrangements allow efficient fumigation of the plants with the terpenoids emitted from the cotton balls. Injection was carried out at the start of the experiment and after 3½ days. In total, 4 mL of the essential oil fraction or limonene were applied to *Arabidopsis* plants per week. 

### 4.3. Real Time PCR

Expression studies were performed to reveal the alterations of *TCP* transcript abundances during the first week of fumigation. The material for qPCR were obtained by harvesting the innermost part of the *Arabidopsis* rosettes with the stem apex and emerging leaves. Each sample comprised 43–51 individual plants per time point, i.e., 1, 3, and 7 days after co-cultivation with *Mentha* plants, application of *Mentha* essential oil and limonene, respectively, and the corresponding controls. The plant material was collected in liquid nitrogen and stored at −80 °C, if not immediately used for RNA extraction. Material from *Brassica rapa* (Chinese cabbage) was likewise harvested after 7 days of fumigation with *Mentha* essential oil or with limonene (Figure 8).

Two replicates were obtained at all three dates, resulting in 6 *Arabidopsis* samples for the treated and control groups. For mRNA preparation, 100 mg of plant material was used. RNA was extracted with the NucleoSpin^®^ RNA Plant” extraction kit (Macherey and Nagel, Düren, Germany) according to the manufacturer’s instructions. RNA quantity was determined by NanoDrop measurements. For cDNA synthesis, the RevertAid First Strand cDNA Synthesis Kit (Thermo Fisher Scientific) was utilized according to the manufacturer’s protocol. 

Real time PCR was performed as described in [44], with the qPCR-Mix “my-Budget 5× EvaGreen^®^ QPCR-Mix II (ROX)” (Bio-Budget Technologies GmbH, Krefeld, Germany) and the thermocycler Applied Biosystems 7300 Real Time PCR System (Thermo Fisher Scientific, München, Germany). For all repetition setups with *Arabidopsis* and for all experiments with Chinese cabbage, the thermocycler CFX Connect (Bio-Rad, Feldkirchen, Germany) was used. Quantitative analysis was carried out for *TCP2*, *TCP3*, *TCP4*, *TCP9*, *TCP10*, *TCP20*, *TCP24*, and *LOX2* expression. Actin (*ACT2*) was used as a reference gene. Primers were synthesized by Eurofins Genomics (Ebersberg, Germany) and are listed in Table S1 [24,45]. The qPCR reaction mixtures (20 µL) contained µL 5× EvaGreen^®^ QPCR-Mix II (ROX, Bio-Budget Technologies GmbH, Krefeld, Germany), 5 µL cDNA sample (15.52 ng/µL), 1 µL forward and reverse Primer Mix (1:1, *v*/*v*; 10 µM each), and 10 µL DNase free H_2_O. For evaluation of RT-qPCR results, the ΔΔCT method was applied.

### 4.4. Determination of Cabbage Dry and Fresh Weights after Fumigation with High Mentha Essential Oil and Limonene Concentrations

For determinations of fresh and dry weights after applications of high amounts of the volatiles, 18-day-old plants were arranged into groups of 24 plants and set up in rows of 4 × 6 under a transparent hood equipped with four cotton balls, as shown in Figure 9. To ensure that the concentration of chemicals was constantly high throughout the experimental period, the cotton balls were soaked every day with either 8 mL limonene or 14 and 16 mL *Mentha* essential oil. Cotton balls of the control groups had no volatile injections. This experiment was performed to elucidate threshold ranges of inhibitory volatile concentrations.

Watering of the plants was performed automatically by an ebb and flow system, which was adjusted to flood the table once a day for 8 min. The heating temperature in the greenhouse was set to 18/15 °C day/night, and the aeration temperature to 21 °C. Trials were started in May and lasted until the end of July. The duration of the treatments was 7 days; afterwards, the plants were cut above ground and fresh weight (FW) and dry weight (DW) after drying at 60 °C for two days were determined. 

### 4.5. Greenhouse Trial with Mentha piperita Plants

**Figure 10:**
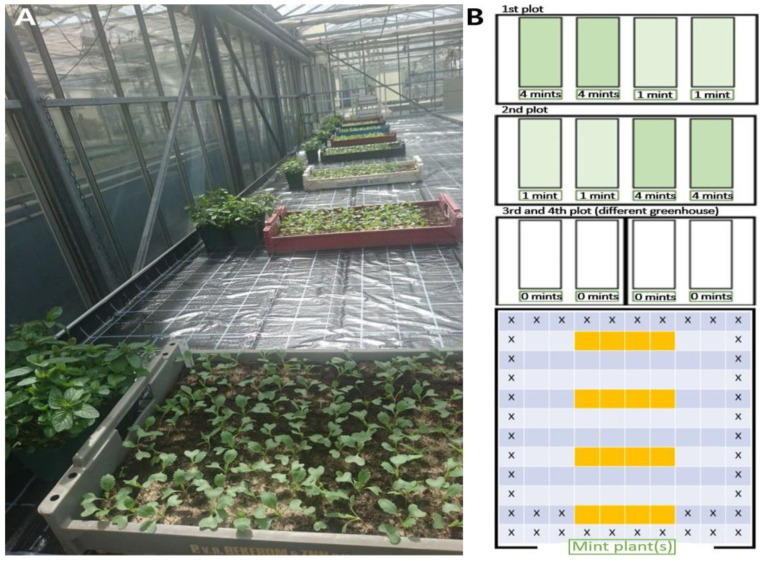
Greenhouse trial. Arrangement of plots (**A**,**B** above) and experimental setup of a single box (8 replications) containing 120 plants (**B** below). The setups in two different greenhouses were carried out in a completely randomized design. Orange box = scored plant (repeated measurements); X = unused plant.

In early May 2018, treatments were started by placing pots with *Mentha* plants next to boxes with 120 ten-day-old white cabbage plants for 21 days (Figure 10). The *Mentha* treatment was run in three plot arrangements with one, four, or no mint plants, the latter as the control. Each plot was placed at a different table with two replicates for each treatment group. The distance between the boxes was 80 cm. The distances of the cabbage plants placed in rows in the boxes to the *Mentha* plants were: 1st row: 5–10 cm; 2nd row: 20–25 cm; 3rd row: 35–40 cm; 4th row: 50–55 cm. Control plants were placed in a neighbored greenhouse and grown under the same conditions (18/15 °C day/night, 16 °C venting temperature, shading when luminous flux was > 60 klx).

Watering was ensured by an automated ebb and flow system (9 min flow, triggered via light integral of 0.6 kWh). Each table (i.e., plot) had its individual immersion pump powering the ebb and flow system. The watering regime was adjusted to 0.4 kWh after four days. The watering conditions for the control plants were correspondingly adjusted. After 11 days, the light integral was changed to 0.9 kWh until the end of the experiment.

### 4.6. Polytunnel Trial

**Figure 11:**
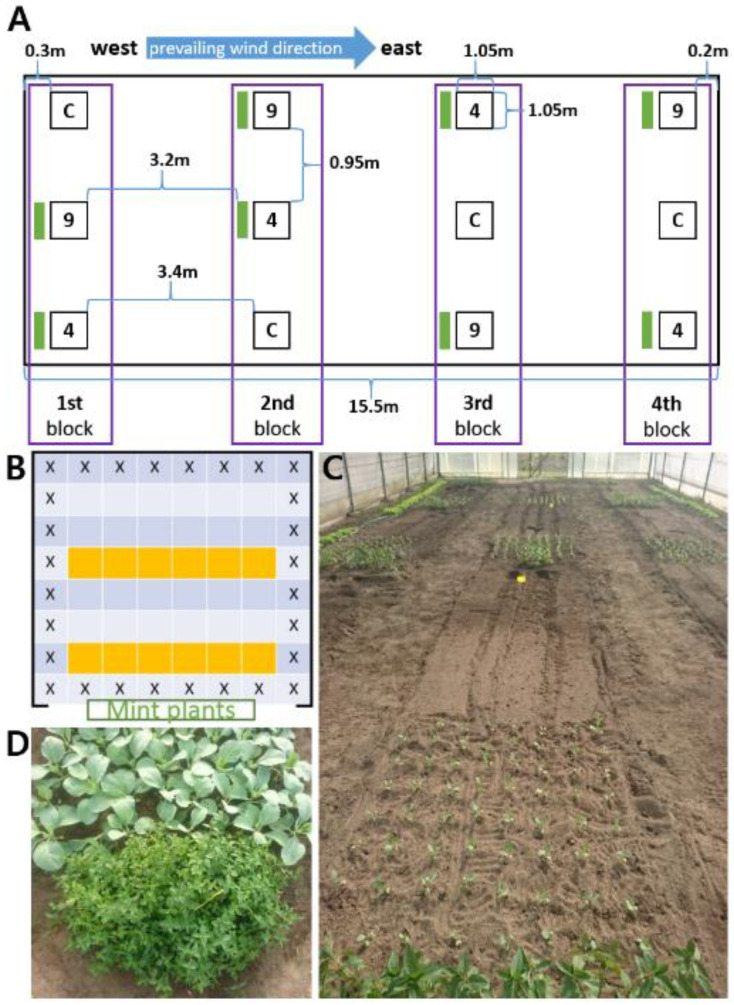
Polytunnel trial. Schema of the experimental setup: total experimental design (**A**) and of a single plot (**B**). Dimensions, the prevailing wind direction, and the positions of individual plots and mints are indicated; (**C**): control plants, 9 and 4: plants exposed to 9 and 4 mints (green bars). orange box = scored plant (repeated measurements); X = unused plant. (**C**): Arrangements of cabbage and *Mentha* plants in the Polytunnel soil; (**D**): picture of a single plot.

Three independent sets were conducted in May and July 2018, following the same design as described for the greenhouse experiments. Four blocks were designed, each consisting of three plots: one untreated control and two co-cultivated plots, here with 4 and 9 mint plants, respectively, taking into account that the air exchange was faster than in the greenhouse. The blocks were arranged as shown in Figure 11, and each block was orthogonally aligned towards the prevailing wind direction. 

*Mentha* plants were placed in front of their respective plots, allowing the air flow to carry the volatiles into the bulk of the cabbage plants. The squarely designed plots had an edge length of 1.05 m with 8 × 8 cabbage plants (Figure 11). Each set was conducted in the same polytunnel. Between the sets, weeds were removed and the roots of the *Mentha* plants were cut to prevent growing towards the experimental plots. In addition, *Mentha* plants were cut to maintain equal size and uniformity. The smallest *Mentha* plant served as reference for the mode of cutting.

After sowing, the young cabbage seedlings of each set were grown for 21 days in a greenhouse, as described above, before planting into the soil covered by the polytunnel. Each set of experiments was run for 22 days before cutting the plants for fresh and dry matter determination. Set 2 was started 8 days after set 1, and set 3 was started 6 days after set 2. 

Watering in the tunnel was carried out by a sprinkler system; once a day for at least 30 min in the first three days after bedding out, then when necessary but at least two to three times a week. 

### 4.7. Measurements of Leaf Parameters

Leaf parameters were determined with leaves longer than 15 mm. Measurement of leaf length and width was performed after 1, 4, 8, 11, and 15 days after exposure to mint volatiles (T1–T5) with plants at 4 different distances (1st row: 5–10 cm; 2nd: 20–25 cm; 3rd: 35–40 cm; 4th: 50–55 cm), and scored as shown in Figure 10. Regarding the polytunnel trial, the leaf parameters length and width were determined for 12 individual plants/plot and at two different distances (1st row: 15–30 cm; 2nd row: 60–75 cm) once a week, 1 (T1), 8 (T3), and 15 (T5) days after the start of co-cultivation (set 1). In the second set, an additional measurement was taken on day 12 (T4) after treatment, and in the third set additional measurements were conducted after 5 (T2) and 12 (T4) days. The methods were the same as described for the greenhouse trial. Evaluations were performed with data obtained from at least 2 plots of identical conditions. Rates of leaf growth increments were determined for T1–T2, T2–T3, T3–T4, and T4–T5. For calculations, zero growth rates were not considered; the quantity of plants/plots showing no increment are mentioned in the text (or Table S1).

### 4.8. Fresh and Dry Weight 

To determine dry matter percentage, plants used for leaf parameter determinations were cut above ground 21 days after treatment. After fresh weight measurements, the plant material was placed in an oven at 105 °C for five days and then used for dry weight determinations. 

### 4.9. Statistics 

All measured variables were subjected to one-way analysis of variance (ANOVA) or to the Kruskal–Wallis test in the case of no Gaussian distribution to verify the differences between the means (GraphPad Software, Inc., La Jolla, CA, USA). Normality was tested according to Anderson–Darling, DÁgostino–Pearson, Shapiro–Wilk, and Kolmogorow–Smirnov. Results are presented in the figures as means + standard error of means. Student’s t-tests were performed in addition and *p*-values provided in the results. 

## Data Availability

Not applicable.

## Supplementary Materials

The following supporting information can be downloaded at: https://www.mdpi.com/article/10.3390/plants11223078/s1, Figure S1: Greenhouse trial leaf growth; Figure S2: Polytunnel trial leaf growth, Table S1: Primer Used for Real Time PCR. Suppliers Volatile Oil Analyses and Certificates.
