# Supplementary material for: Modulating Expression Levels of TCP Transcription Factors by Mentha x piperita Volatiles—An Allelopathic Tool to Influence Leaf Growth?
Source: Plants (Basel). 2022 Nov 14;11(22):3078. doi: 10.3390/plants11223078 (PMC9697212; doi:10.3390/plants11223078)
Supplement: Supplementary file 1 [file plants-11-03078-s001.zip › plants-1995274-supplementary.pdf]

Supplementary File to

Modulating Expression Levels of TCP Transcription Factors by *Mentha x piperita* Volatiles – An Allelopathic Tool to Influence Leaf Growth?

Matthias Preusche[1,2], Marvin Vahl[1], Johanna Riediger[1,2], Andreas Ulbrich[1] and Margot Schulz[2]

[1] Affiliation 1 Department of Horticultural Production, University of Applied Science, Osnabrück, Germany; m.preusche@hs-osnabrueck.de; a.ulbrich@hs-osnabrueck.de; m.vahl@hs-osnabrueck.de

[2] Affiliation 2 Institute of Molecular Physiology and Biotechnology of Plants (IMBIO), University of Bonn; ulp509@uni-bonn.de

*****Correspondence: ulp509@uni-bonn.de

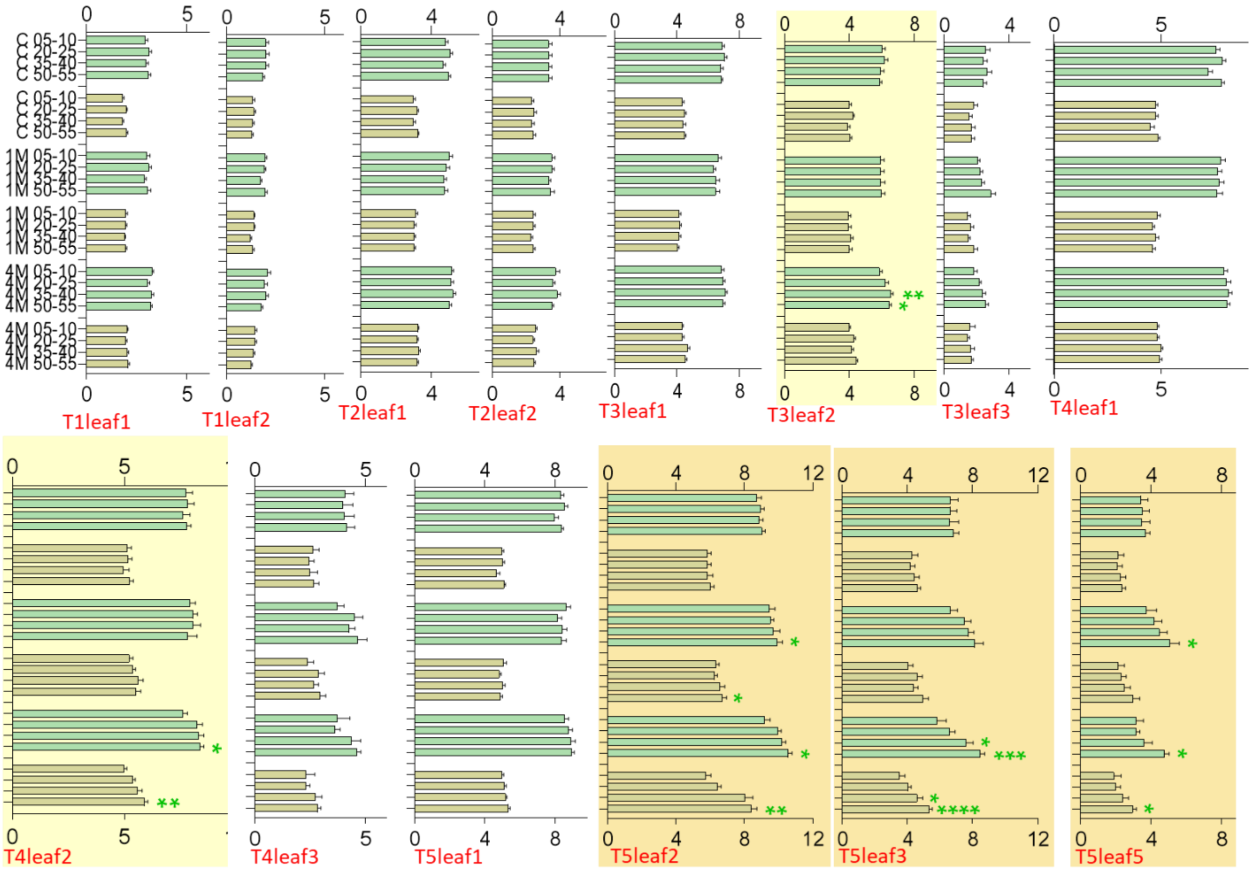

Figure S1. Greenhouse trial. White cabbage leaf length (green) and width growth (olive) of the control (C) in presence of 1 (1M) or 4 *Mentha* plants (4M) at the distances 05-10, 20-25, 35-40 and 50-55 cm. Growth parameters are shown for single leaves. T1: leaf 1 and leaf 2; T2: leaf 1 and leaf 2; T3: leaf 1, 2, and 3; T4: leaf 1, 2, and 3; T5: leaf 1, 2, 3, and 4. Colored backgrounds mark leaves with the highest responsiveness to the treatment. At T1, no response to *Mentha* volatiles was found. The highest response was developed at the latest phase of the fumigation. Green asterisks indicate increase, significance levels: (t-test): *$p < 0.05$; **$p < 0.005$; ***$p < 0.0005$); bars without asterisk: no significance.

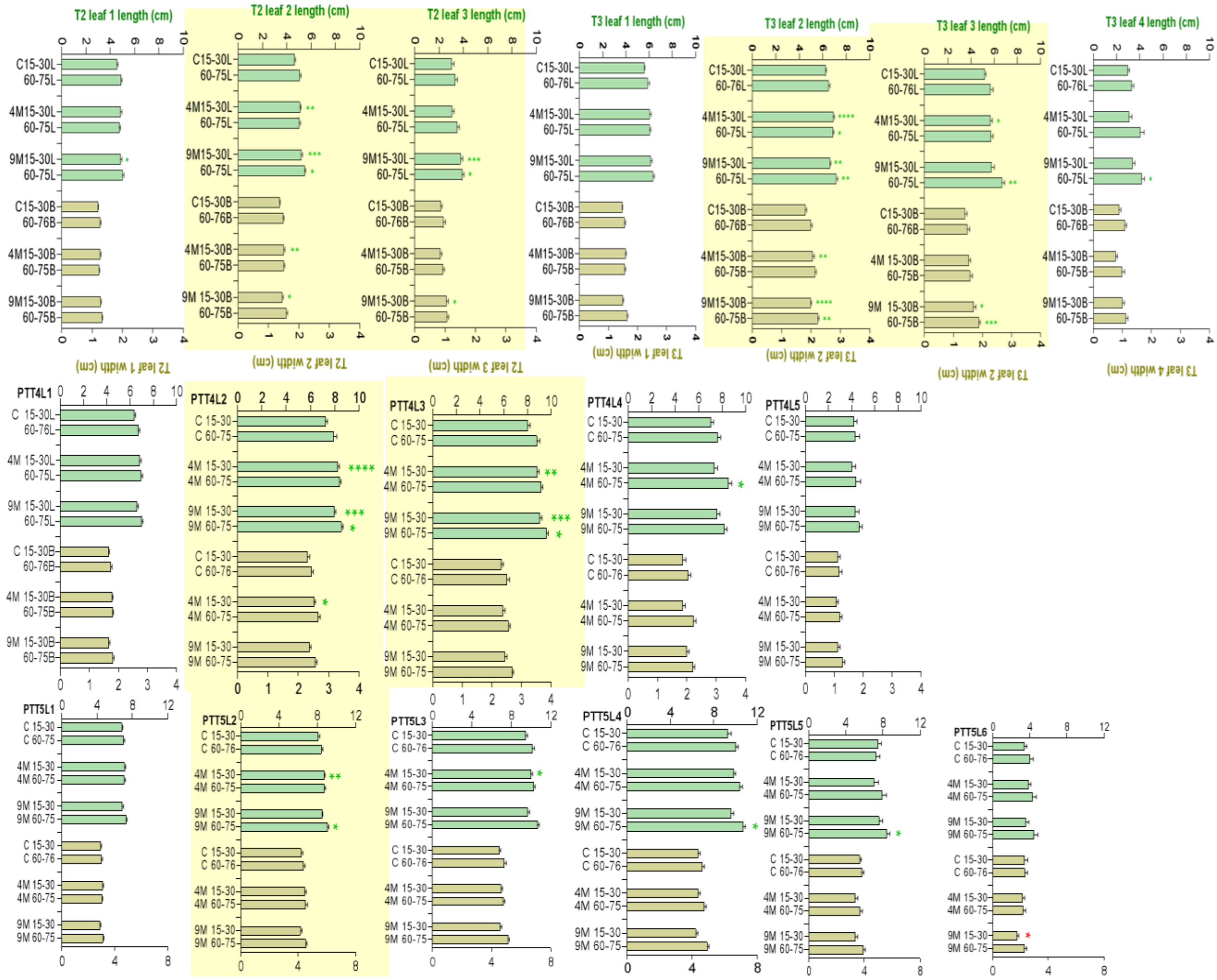

Figure S2. Polytunnel Trial: White cabbage leaf length (green) and width growth (olive) of the control (C) in presence of 4 (4M) or 9 *Mentha* plants (9M) at the distances 15-30 and 55-75 cm. Growth parameters are shown for single leaves. T2: leaf 1, 2 and 3; T3: leaf 1, 2, 3, and 4; T4: leaf 1, 2, 3, 4 and 5, T5: leaf 1, 2, 3, 4, 5 and 6. Light yellow backgrounds mark leaves with the highest responsiveness to the treatment. Green asterisks indicate increase, red asterisk significant lower growth compared to the controls, significance levels: (t-test): *p < 0.05; **p < 0.005; ***p < 0.0005); bars without asterisk: no significance.

Table S1. Primer Used for Real Time PCR. F: forward Primer; R: reverse Primer
Compare [**19, 41**] references main text.

Arabidopsis thaliana	
ACT2-F	CAT CAT CAT CCT CAT CAT CAG CAT CA
ACT2-R	AGA TTG AAT GCT CCG CCG TTG GA
LOX2-F	GAT GCC CCA GTT CTC ATT AAC AGG G
LOX2-R	CGG GTC TAG TTT GCT TAT TAA CGG C
TCP2-F	CAT CAT CAT CCT CAT CAT CAG CAT CA
TCP2-R	AGA TTG AAT GCT CCG CCG TTG GA

TCP3-F	ACC TAT GAT TCG TGC TTG GTT TGA T
TCP3-R	GAG GGA TAT GGT AGG GAT GAT GAT G
TCP4-F	TTC GGA AGG ATT CAG AGA CTA GTG G
TCP4-R	AGG AGT AGG AGG AGC GAA CAG AAA C
TCP9-F	TGC CGT CGT TTT CAA TGT CTT TAG C
TCP9-R	TTC TCC CTC TTC CTT CAA CCT TCG
TCP10-F	AAG CTT CTT CGA TGT TTG CTT CAT C
TCP10-R	GCT TCT TCT CCG TGG AGT AGT CTT G
TCP20-F	TTA GGC TTG TCT CAA GAA GGG AAT G
TCP20-R	GCA TAT GGT GAA GAA CCC TAC CTT G
TCP24-F	CCT TCA GTC CAA TTC ACA ATC TCT CT
TCP24-R	GCT TTG ACT ATC CAT TGG AGA AGA G
ACT2-F	CTTGCACCAAGCAGCATGAA
ACT2-R	CCGATCCAGACACTGTACTTCCTT
Brassica rapa	
BrTCP2aF	GTGCTCACCCTTTCTCCGAC
BrTCP2aR	ATCCTGCCGGAGAAATCAGA
BrTCP2bF	TCCGATGAACGATGACGACA
BrTCP2bR	GACAACGAGTTAGCCGCAGC
BrTCP3F	GCGAGATCGTGGAGGTTGAA
BrTCP3R	TGGCCGTACAGACTTTGCTG
BrTCP4aF	GTTCTACGGGCAGAGCAATCC
BrTCP4aR	GCCACCAGCCTCTGAATTGA
BrTCP4bF	TTCTTCTTCAATGAGACGCCG
BrTCP4bR	TGACCTCCTTGCACCTCGAC
BrTCP4cF	GACTGGTGGCTTGTAACGGC
BrTCP4cR	TCGTCGGAGAAGGAGCAAAG
BrTCP9aF	CGGCCATCGCTATGTCTGTT
BrTCP9aR	CCGAGTTATCTCCCACGTCAG
BrTCP9bF	CTCCACCGTCGTCGTCAATA
BrTCP9bR	TTCCTCTTCCTTCGACCTTGG
BrTCP10F	GTTATAGCGCCGGAGATGGA
BrTCP10R	GGTCTTTTCGTCCTGTGGCTC
BrTCP20aF	TTTTGCGTCCATTCTTGGTG
BrTCP20aR	CCAACATTCCCTTCCTGAGC
BrTCP20bF	GTAAGAAGCAGCTTGCCCCC
BrTCP20bR	TCCTGATTCTCCGACCGC
BrTCP24aF	CAAGACCGTCTTGGCTTCGA
BrTCP24aR	CAACTCGGAGATGGAGTCGG
BrTCP24bF	GCGGCTTCTGACTCAATCTCC
BrTCP24bR	CGTGCTCGACTTAGACAGCG
BrActin3F	GTACAACCGGCATCGTGCTT
BrActin3R	CATGTGGAAGTGCATAACCCTC
